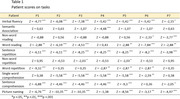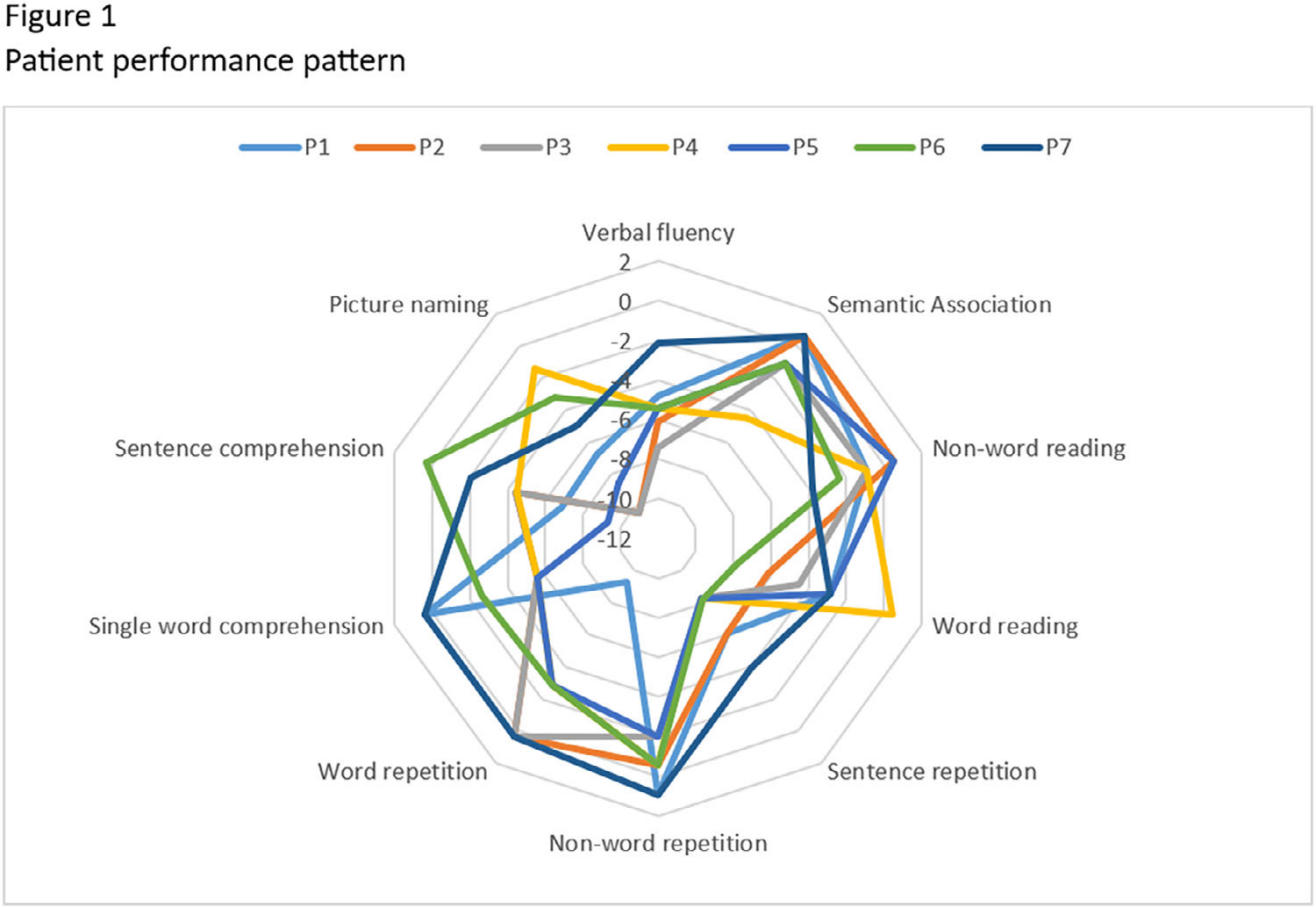# Symptomatic diversity in the logopenic variant of Primary Progressive Aphasia

**DOI:** 10.1002/alz70857_106008

**Published:** 2025-12-25

**Authors:** Axel Fernández‐Zaionz, Leticia Vivas

**Affiliations:** ^1^ Institute of Basic and Applied Psychology and Technology (IPSIBAT), National University of Mar del Plata (UNMDP), Psychology Faculty, Mar del Plata, Buenos Aires, Argentina; ^2^ National Council of Scientific and Technical Research (CONICET), Mar del Plata, Buenos Aires, Argentina

## Abstract

**Background:**

While there is a well‐established core profile that allows the classification of the logopenic variant of Primary Progressive Aphasia (PPA), including sentence repetition impairment, naming impairment, and spared single‐word comprehension (Gorno‐Tempini et al., 2011), clinical practice shows that the symptomatic profile can be quite variable. Several authors have described this variability in symptoms (Macoir et al, 2021), and there is also evidence that points toward identifying patterns within this variability and even identifying mixed variants with unique clinical characteristics (Mazzeo et al., 2024). This study aims to contribute to the identification of such profiles.

**Method:**

Sample: 7 patients with the early‐stage logopenic variant of PPA, matched with 90 healthy controls. Procedure: The Argentine version of the Mini Linguistic State Examination was administered. The Singlims_ES software (Crawford & Garthwaite, 2002) was used to analyze whether the task performance differences were statistically significant. Subsequently, individual profiles were compared to identify commonalities.

**Result:**

Profiles consistent with the core features of lvPPA were identified. Participants P1 and P7 showed severe impairment in sentence repetition, sentence comprehension, picture naming, and verbal fluency, with good performance in single‐word comprehension and semantic association, while P2, P3, and P5 exhibited a similar pattern, albeit with clear deterioration in single‐word comprehension (Figure 1). Participant P6 presented a similar profile, except for good sentence comprehension performance. On the other hand, markedly diverse performance patterns compared to the typical lvPPA profile were found, such as in the case of P4 (Figure 1). P4 stood out due to significant impairment in both semantic association and single‐word comprehension, with relatively preserved picture naming and good word‐reading performance.

**Conclusion:**

While profiles with the core characteristics of lvPPA were identified, discrepancies among participants, such as the differences in performance between P1, P2, and P6, highlight the symptomatic diversity within lvPPA. Additionally, participants like P4, despite retaining the core characteristics of lvPPA, exhibited secondary features more aligned with semantic deficits, suggesting the existence of mixed profiles, as proposed by Mazzeo et al. (2024). These findings underscore the importance of detailed profile analysis to improve diagnostic accuracy in PPA.